# Resolving Discrepant Findings on ANGPTL8 in β-Cell Proliferation: A Collaborative Approach to Resolving the Betatrophin Controversy

**DOI:** 10.1371/journal.pone.0159276

**Published:** 2016-07-13

**Authors:** Aaron R. Cox, Ornella Barrandon, Erica P. Cai, Jacqueline S. Rios, Julia Chavez, Claire W. Bonnyman, Carol J. Lam, Peng Yi, Douglas A. Melton, Jake A. Kushner

**Affiliations:** 1 McNair Medical Institute, Pediatric Diabetes and Endocrinology, Baylor College of Medicine, Texas Children’s Hospital, Houston, Texas, United States of America; 2 Stem Cell and Regenerative Biology, Harvard Stem Cell Institute, Harvard University, Cambridge, Massachusetts, United States of America; 3 Joslin Diabetes Center, Harvard Medical School, Boston, Massachusetts, United States of America; University of British Columbia, CANADA

## Abstract

The β-cell mitogenic effects of ANGPTL8 have been subjected to substantial debate. The original findings suggested that ANGPTL8 overexpression in mice induced a 17-fold increase in β-cell proliferation. Subsequent studies in mice contested this claim, but a more recent report in rats supported the original observations. These conflicting results might be explained by variable ANGPTL8 expression and differing methods of β-cell quantification. To resolve the controversy, three independent labs collaborated on a blinded study to test the effects of ANGPTL8 upon β-cell proliferation. Recombinant human betatrophin (hBT) fused to maltose binding protein (MBP) was delivered to mice by intravenous injection. The results demonstrate that ANGPTL8 does not stimulate significant β-cell proliferation. Each lab employed different methods for β-cell identification, resulting in variable quantification of β-cell proliferation and suggests a need for standardizing practices for β-cell quantification. We also observed a new action of ANGPTL8 in stimulating CD45^+^ hematopoietic-derived cell proliferation which may explain, in part, published discrepancies. Overall, the hypothesis that ANGPTL8 induces dramatic and specific β-cell proliferation can no longer be supported. However, while ANGPTL8 does not stimulate robust β-cell proliferation, the original experimental model using drug-induced (S961) insulin resistance was validated in subsequent studies, and thus still represents a robust system for studying signals that are either necessary or sufficient for β-cell expansion. As an added note, we would like to commend collaborative group efforts, with repetition of results and procedures in multiple laboratories, as an effective method to resolve discrepancies in the literature.

## Introduction

The identification of a liver-derived protein with β-cell mitogenic properties, which was re-named betatrophin to draw attention to its effect on pancreatic β-cell replication, initially produced interest, and subsequent controversy concerning its purported mitogenic properties. After administering an insulin receptor antagonist (S961), Yi et al. observed a significant elevation in liver expression of the above mentioned betatrophin gene [also called ANGPTL8 [1], lipasin [2], and RIFL [3]; which we will hereafter refer to as ANGPTL8] and its expression was intriguingly associated with β-cell proliferation in the setting of the induced-insulin resistance phenotype [4]. Yi et al. overexpressed ANGPTL8 cDNA in the liver of mice via hydrodynamic tail vein injection and reported that ANGPTL8 overexpression induced a striking 17-fold increase in β-cell proliferation. These findings pointed to ANGPTL8 as a powerful β-cell mitogen potentially useful as the basis for a therapy.

Subsequent studies raised doubts about these initial ANGPTL8 observations. *Anpgtl8* knockout mice exhibited normal glucose homeostasis, β-cell area, and compensatory β-cell expansion when challenged with a high fat diet [5, 6]. These results suggested that *Angptl8* is dispensable for normal and regenerative β-cell growth. Gromada and colleagues [6] further reported that overexpression of human *ANGPTL8* in the liver of mice did not alter β-cell area, in direct contrast to the report by Yi et al [4].

A comprehensive study by Kushner and colleagues aimed to further interrogate the controversy surrounding the betatrophin hypothesis [7]. Overexpression of *Angptl8* was performed in multiple cohorts of young and aged mice of various mouse genetic strains. Compared to controls, *Angplt8* overexpression had no effect on β-cell proliferation in any of the five cohorts tested, with no change in β-cell mass. These observations support and extend the results of the Gromada group [6], and strongly suggested that ANGPTL8 is not a β-cell mitogen.

There were concerns regarding the highly variable expression of *Angplt8* in the liver resulting from the technically challenging and inefficient system for delivery of *Angptl8* cDNA. Other delivery methods could improve administration of ANPTL8, for example, Grayburn and colleagues used ultrasound targeted microbubble destruction to deliver human *ANGPTL8* plasmids to the rat pancreas [8]. This *ANGPTL8* treatment resulted in a 7-fold increase in β-cell proliferation versus control in young rats. While species-specific responses to ANGPTL8 may vary, these observations again provided support to the original betatrophin hypothesis, suggesting that improved methods of ANGPTL8 delivery to the pancreas could be used to effect significant β-cell proliferation.

Our current objective is to provide a resolution to the betatrophin hypothesis and thus the mechanism of action of ANGPTL8 by working collaboratively on a blinded study where one directly assesses the experimental basis of the aforementioned discordant results. We administered recombinant human betatrophin (hBT) fused to maltose binding protein (MBP) by intravenous injection as a direct method for consistent systemic delivery of highly soluble ANGPTL8, as an alternative to the indirect method of increasing ANPTL8 levels through plasmid DNA injection.

Independent blinded analysis of β-cell proliferation by our three different research groups (Kushner, Melton and Yi) revealed inter-group variability in identifying β-cells and in quantifying their replication. Nonetheless, collectively the results show that ANGPTL8 does not stimulate robust β-cell proliferation. Moreover, we found that ANGPTL8 treatment in mice stimulated hematopoietic-derived cell proliferation within the islet, as well as extra-islet cell proliferation, and this may have confounded measurements that contributed to the variability amongst labs. In aggregate, the hypothesis that ANGPTL8 induces dramatic and specific β-cell proliferation is not supported by our improved tests and analysis. We determined that the participating laboratories were using methods that resulted in consistently biased cell enumeration. Therefore, in addition to our negative results concerning the betatrophin hypothesis, this report calls attention to the fact that methodologies for enumerating β-cell numbers and their replication rates need to be standardized across laboratories. Such standardization, by its very nature, requires the collaborative efforts of many interacting laboratories.

## Materials and Methods

### Collaborative Blinded Study

**Fig 1:**
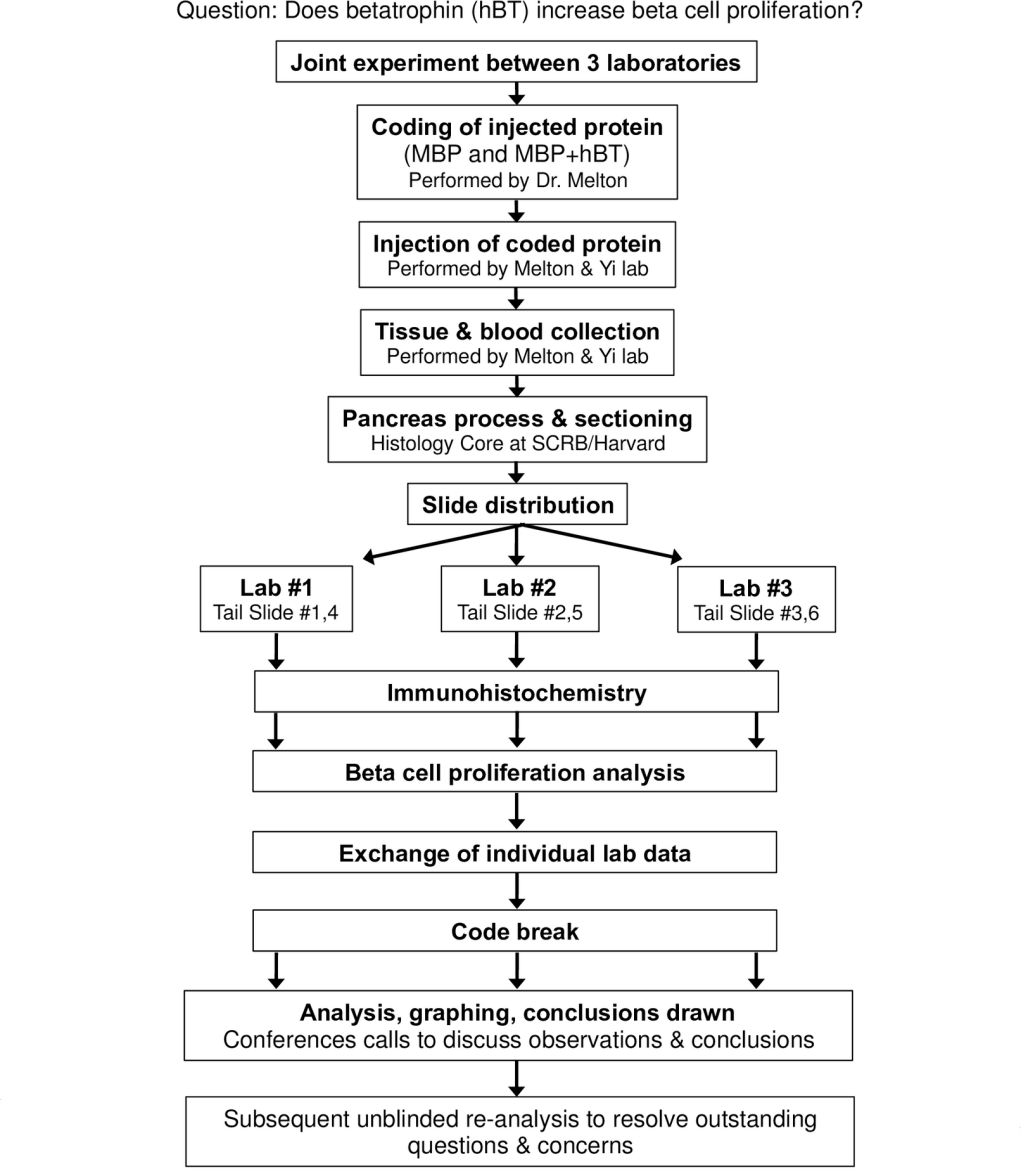
Schematic design of a collaborative, blinded experiment.

Three laboratories participated in this blinded study: Lab #1 –Dr. Melton, Lab #2 –Dr. Kushner, Lab #3 –Dr. Yi. The experimental design is described in Fig 1.

### Mice

This study was carried out in strict accordance with the recommendations in the Guide for the Care and Use of Laboratory Animals of the National Institutes of Health. The protocol was approved by the Harvard University Animal Care and Use Committee. All efforts were made to minimize suffering. Ten-week-old CD1 male (Hsd:ICR [CD-1]) mice were from Harlan Labs (Indianapolis, IN). Mice were fed Rodent NIH-31 M 18–5 Auto diet (Colonial Scientific Inc., Richmond, VA) and housed with a standard light:dark cycle. Mice were labeled continuously via the drinking water with 5-ethynyl-2’-deoxyuridine (EdU, 0.5 g/L; Life Technologies, Grand Island, NY). Mice were sacrificed on day 3 for pancreatic morphometric analysis. Fed serum samples were analyzed with Mouse Ultrasensitive Insulin ELISA (Alpco, Salem, NH), ANGPTL8 mouse ELISA kit (SEW803Mu; Cloud-Clone Corp., Houston, TX), human betatrophin ELISA kit (EK-051-60; Phoenix Pharmaceuticals, Inc., Burlingame, CA), and Triglyceride Colorimetric Assay Kit (#10010303; Cayman Chemical, Ann Arbor, MI).

### Plasmid Expression Vectors

cDNA encoding human ANGPTL8 (hBT) protein without the signal peptide (a.a. 22–197) was amplified by PCR. Maltose binding protein (MBP) (a.a.1-367) was produced using pMal-c5x vector (New England BioLabs Inc., Ipswich, MA). Plasmid encoding MBP+hBT protein was a gift from Evotec A.G. (Hamburg, Germany). A TEV protease cleave site was inserted between MBP and hBT. The plasmids were transformed into NEB express *E*.*coli* ER2523 (New England BioLabs Inc.) for protein expression.

### Recombinant Protein Expression

Both MBP and MBP+hBT recombinant protein were produced according to the instruction of pMAL Protein Fusion & Purification System (New England BioLabs). Maltose in the elution was removed by dialysis. Maltose-free MBP or MBP+hBT protein was quantified (OD 280 nm). Dialyzed protein was snap frozen, stored at -80^°^C.

### Western Blot

MBP and MBP+hBT proteins were immunoprecipitated from bacterial lysates and mouse serum samples by Amylose resin, MBP antibody (New England BioLabs), or human Betatrophin antibody (Phoenix Pharmaceuticals). Two μg of protein were run on 4–15% SDS-PAGE gel and transferred to nitrocellulose membrane. Coomassie blue staining was used to identify MBP (42 kDa) and MBP+hBT (64 kDa).

### Tail Vein Injection

Eleven μg MBP or MBP+hBT protein in 200 μl of 0.2 μm membrane filtered column buffer were tail vein injected on day 1 and 2. Mice were placed in a restrainer under a heating device to dilate the tail vein. The injection was performed within 3 seconds using a 27-gauge needle.

### Immunohistochemistry

Pancreas head and tail paraffin sections (5μm thick) as previous [9, 10]. Pancreas tail was sectioned (pancreas head was saved) for distribution with every third section on a separate slide, and 200 μm between levels. Each lab received sections that were adjacent to those analyzed by the 2 other labs; a slide sent to Lab#1 for analysis might have sections 1, 4, 7, 10, etc. and Lab#2 would receive a slide with sections 2, 5, 8, 11 from the same pancreas. Lab #1 primary antisera included polyclonal guinea pig anti-insulin (#A0564; Dako, Carpinteria, CA) and monoclonal rabbit anti-Ki67 SP6 (#ab16667; Abcam, Cambridge, MA) with donkey secondary antisera conjugated to Cy2 or Cy3 (#A21206, #A21203; LifeTechnology, Carlsbad, CA). Lab #2 primary antisera included polyclonal guinea pig anti-insulin (#A0564; Dako), monoclonal mouse anti-human Ki67 (#550609; BD Biosciences, San Jose, CA), monoclonal mouse anti-Nkx6.1 (#F55A12-c; Developmental Studies Hybridoma Bank, Iowa City, Iowa), polyclonal rabbit anti-glucagon (#ab8055; Abcam), polyclonal rabbit anti-synaptophysin (#180130; ThermoFisher Scientific, Waltham, MA), monoclonal mouse anti-CD3 (#ab17143; Abcam), rat anti-mouse B220 (#557390; BD Biosciences), monoclonal rat anti-F4/80 (#ab16911, Abcam), monoclonal Armenian hamster anti-CD11c (#MCA1369GA; AbD Serotece, Raleigh, NC), monoclonal rat anti-mouse Gr-1 (#550291; BD Biosciences), monoclonal rat anti-E-cadherin (#13–1900; ThermoFisher), mouse anti-N-cadherin (#610920; BD Biosciences), monoclonal mouse anti-smooth muscle actin alpha (#IS611; Dako), polyclonal rabbit anti-desmin (#PA1-37556; ThermoFisher), polyclonal rabbit anti-CD31 (#ab28364; Abcam), rat anti-CD34 (#553731; BD Biosciences), and rat anti-CD45 (#550539; BD Biosciences). Donkey or goat secondary antisera were conjugated to Cy2, Cy3, or Cy5 (#706-166-148, #711-165-152, #715-605-151, #127-605-160, #127-545-160; Jackson ImmunoResearch Laboratories, West Grove, PA), incubated with DAPI (Molecular Probes, Eugene, OR). Lab #3 primary antisera included polyclonal guinea pig anti-insulin (#A0564; Dako) and monoclonal rabbit anti-Ki67 SP6 (#ab16667; Abcam) with goat secondary antisera conjugated to Alexa Flour 488 or 594 (#A11073, #A21207; LifeTechnology). EdU was detected with Click-iT EdU Alexa Fluor 555 or 647 kit according to the manufacturer’s protocol (ThermoFisher).

### Proliferation Analysis

Every islet was imaged within 6–8 sections across 2 slides by each lab. Lab #1 imaged with the 20x objective (numerical aperture (NA) = 0.8) and the 10x eyepiece of the Zeiss Imager Z2 microscope (Carl Zeiss MicroImaging, Thornwood, NY) for quantification of β-cell proliferation. Both EdU^+^ insulin^+^ DAPI^+^ cells and Ki67^+^ insulin^+^ DAPI^+^ cells were counted manually using the multi-point tool of the ImageJ software. An average of 3,040 (ranging from 941 to 6,124) β-cells were counted. Ki67^+^ or EdU^+^ β-cell ratios were calculated as percent of total insulin positive cells. Lab #2 imaged with a 40x objective (NA = 0.75), Zeiss Axio Imager (Carl Zeiss MicroImaging) for quantification of β-cell proliferation as previously described [9]. Ki67^+^ insulin^+^ DAPI^+^ cells were counted by hand, due to the high background of the mouse Ki67 primary antibody. EdU^+^ insulin^+^ DAPI^+^ cells were counted using Volocity (PerkinElmer, Waltham, MA). An average of 6,900 (ranging from 1,553 to 11,023) insulin^+^ DAPI^+^ cells were counted in the total pancreas for Ki67 and EdU. In a subsequent analysis using Nkx6.1 to better identify β-cells, an average of 8,400 (ranging from 2,405 to 15,043) Nkx6.1^+^ insulin^+^ DAPI^+^ were counted for Ki67 and EdU using Volocity. Ki67^+^ or EdU^+^ β-cell ratios were calculated as percent of total insulin positive (Nkx6.1 positive) cells. Extra-islet cell proliferation determined by number of EdU^+^ or Ki67^+^ cells outside of the islet in the exocrine space, as a percentage of total non-islet cells. CD45^+^ cells were identified within the intra- and peri-islet region by dilating insulin area by one cell’s diameter and filling all holes within the object; the percentage of these cells expressing EdU was quantified for CD45 cell proliferation. Lab #3 imaged with the 10x objective (NA = 0.3) and 10x eyepiece of the Life Technology EVOS FL Auto microscope for quantification of β-cell proliferation. Both EdU^+^ insulin^+^ DAPI^+^ cells and Ki67^+^ insulin^+^ DAPI^+^ cells were counted using a Matlab based script. An average of 3,705 (ranging from 897 to 10,914) β-cells defined by DAPI^+^ nucleus in insulin^+^ region, were counted in the total pancreas for Ki67 and an average of 3,390 (ranging from 450 to 9,402) β-cells for EdU.

### Statistics

All results are reported as mean ± standard error. Statistical analysis was performed with an ANOVA (one-way, two-way, or repeated measures) or a student’s t-test (unpaired) as indicated, and reported as *p* values.

## Results

### A collaborative, blinded approach to resolve a controversial topic

**Fig 2:**
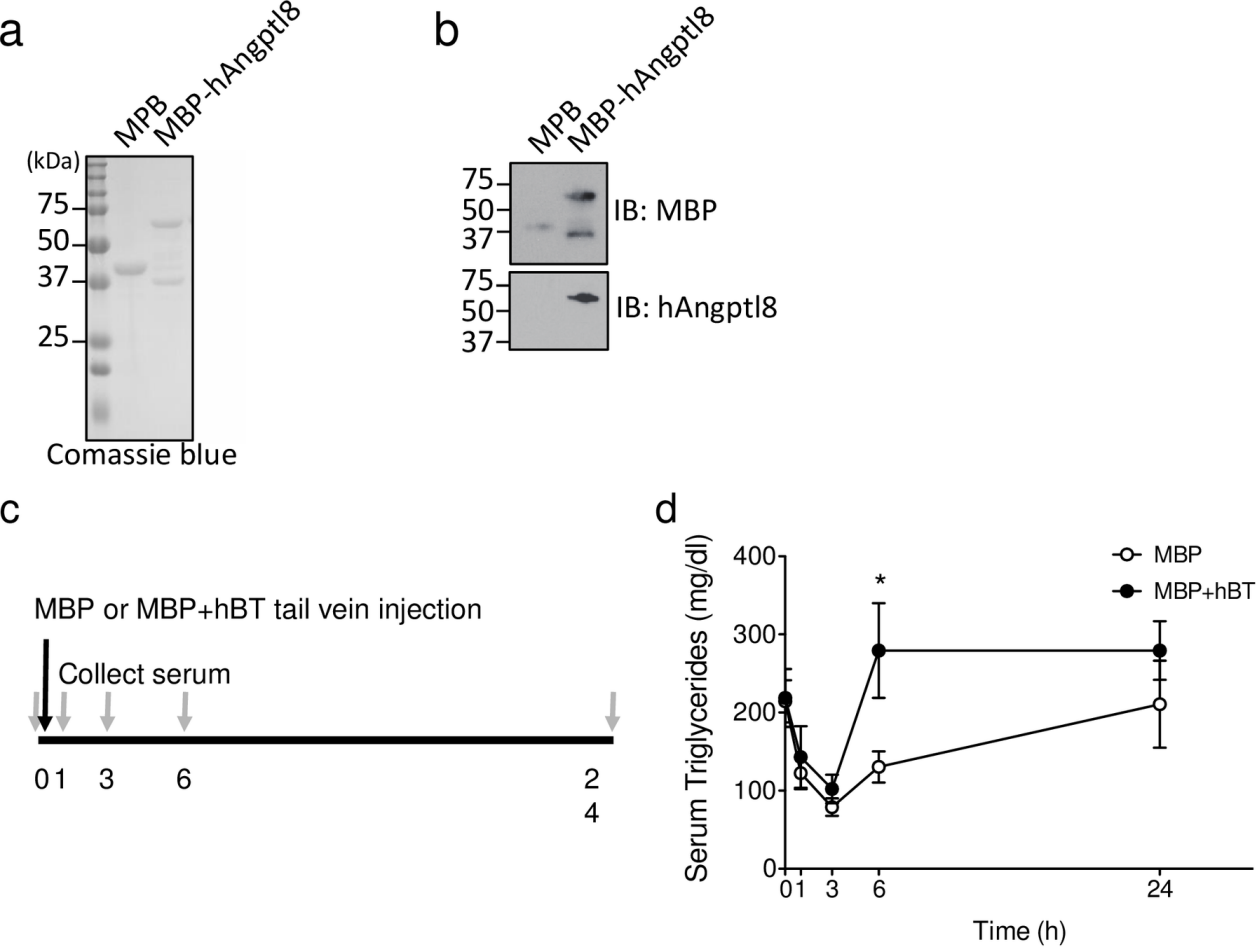
ANGPTL8 is detected in serum and acutely increases triglycerides. **(a-b)** SDS-PAGE gel electrophoresis of cell lysates from MBP and MBP+hBT expressing *E*.*coli* cells with **(a)** Coomassie blue staining and **(b)** western blot detection of MBP (42 kDa) and hBT (64 kDa). **(c)** Timing of serum collection and MBP or MBP+hBT tail vein injection. **(d)** Serum triglyceride (mg/del) levels over time (h). Data are reported as the mean ± SEM. MBP and MBP+hBT = 5 animals per group. Two-way ANOVA with a Bonferroni posthoc test was performed. * *p* < 0.05 versus MBP.

**Fig 3:**
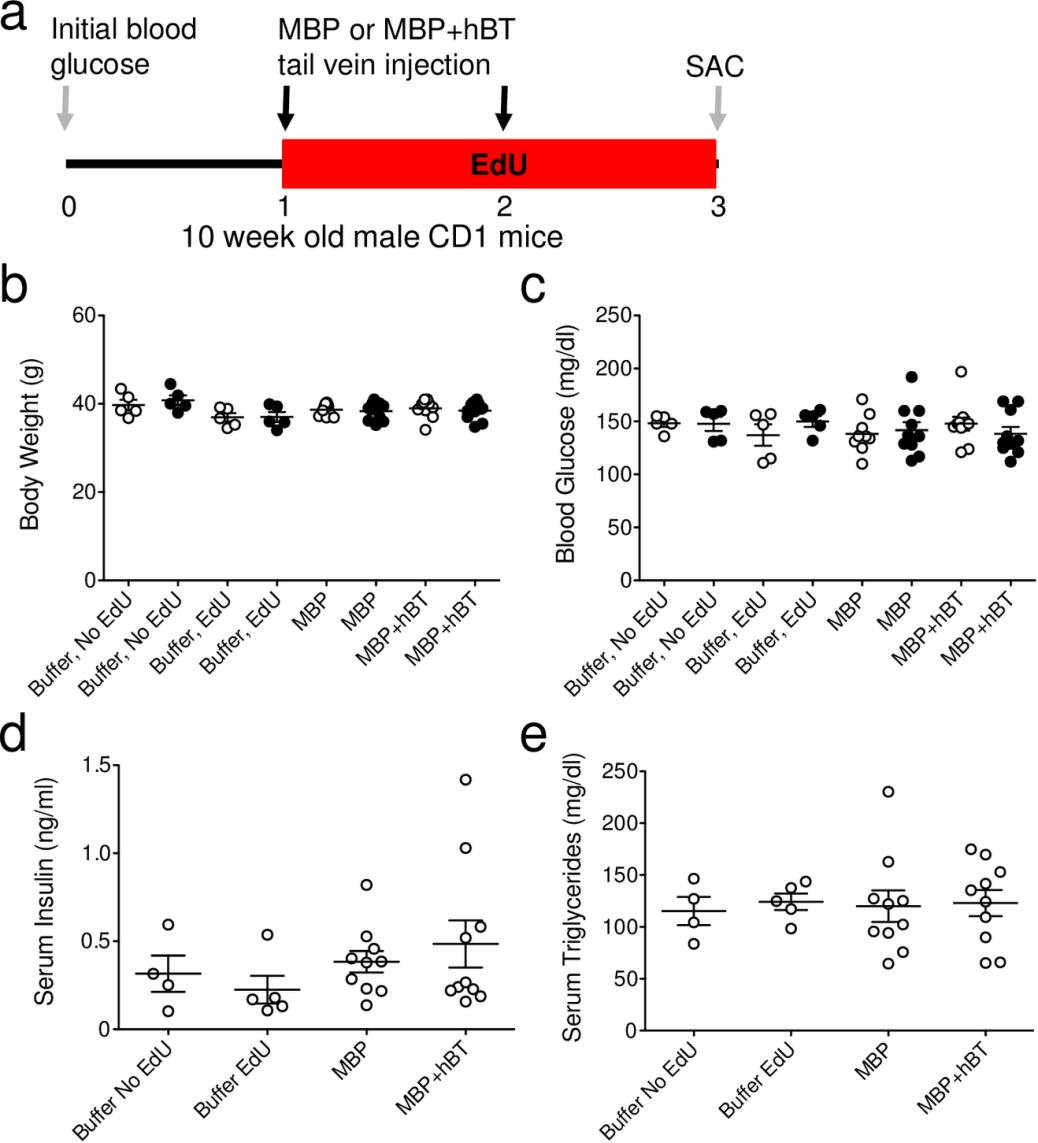
ANGPTL8 treatment is well tolerated in mice. **(a)** Timeline indicating blood glucose sampling, EdU labeling, tail vein injection of MBP or MBP+hBT on day 1 and 2, with sacrifice on day 3. **(b-c)** Initial (white circles) and final (black circles) **(b)** body weight (g) and **(c)** blood glucose (mg/dl). Two-way ANOVA was performed. **(d-e)** Random fed serum **(d)** insulin (ng/ml) and **(e)** triglycerides (mg/dl). Data are mean ± SEM. Buffer, no EdU and buffer, EdU = 4–5 animals per group; MBP and MBP+hBT = 10 animals per group. One-way ANOVA was performed.

**Fig 4:**
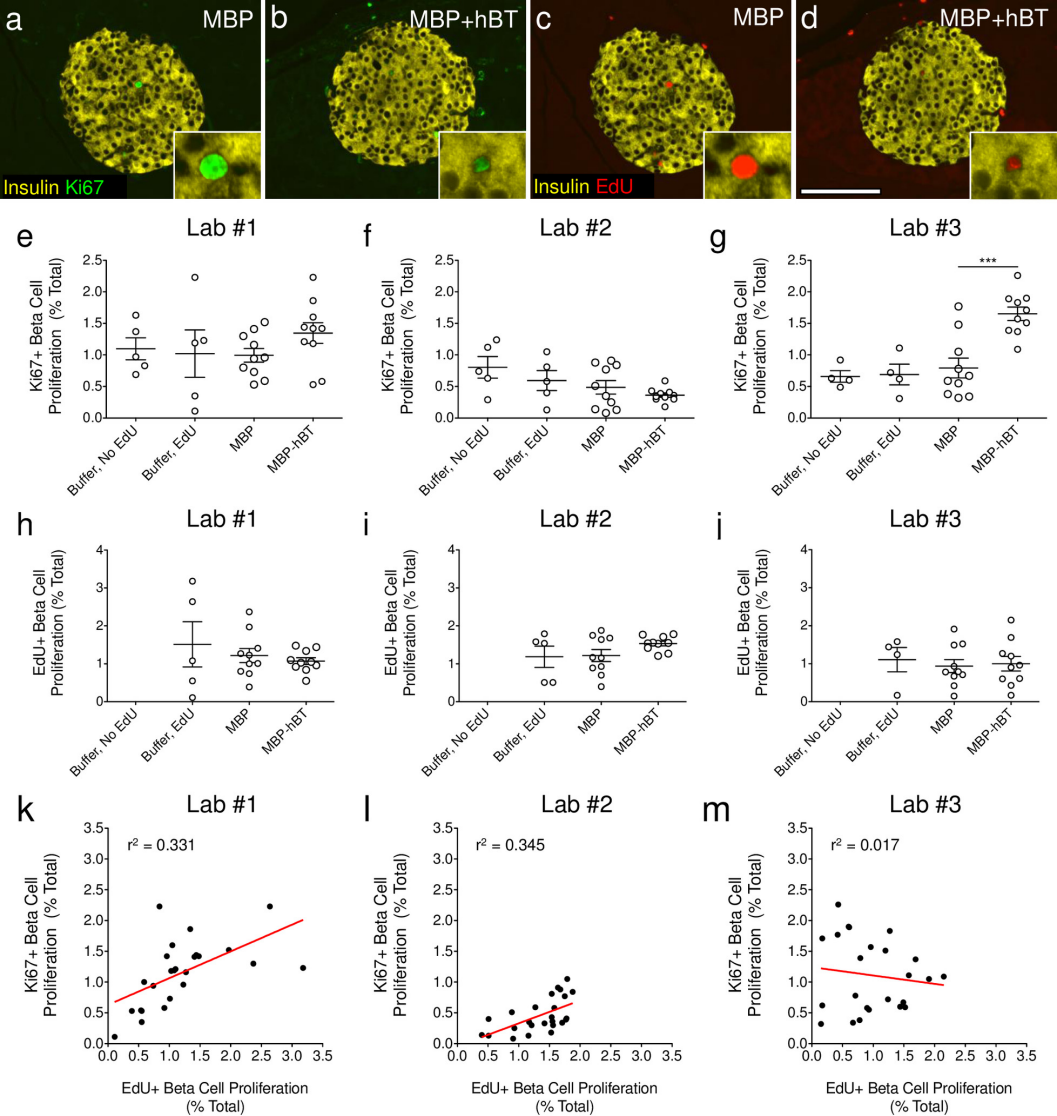
ANGPTL8 treatment in mice has no effect on β-cell proliferation. **(a-d)** Staining for insulin (yellow) and **(a-b)** Ki67 (green) or **(c-d)** EdU (red) for **(a,c)** MBP and **(b,d)** MBP+hBT islets. Scale bar: 100 μm. **(e-j)** β-cell proliferation quantified by insulin^+^ DAPI^+^ cells containing **(e-g)** Ki67 or **(h-j)** EdU as a percentage of total β-cells, from two control groups (buffer injection alone or with EdU), MBP, and MBP+hBT samples. One-way ANOVA was performed with Bonferroni’s multiple comparison test. *** *p* < 0.001 MBP+hBT versus MBP, ** *p* < 0.01 MBP+hBT versus Buffer, no EdU and buffer, EdU. **(k-m)** Linear regression analysis of the correlation between EdU^+^ and Ki67^+^ β-cell proliferation. Data are mean ± SEM. Buffer, no EdU and buffer, EdU = 5 animals per group; MBP and MBP+hBT = 10 animals per group.

**Fig 5:**
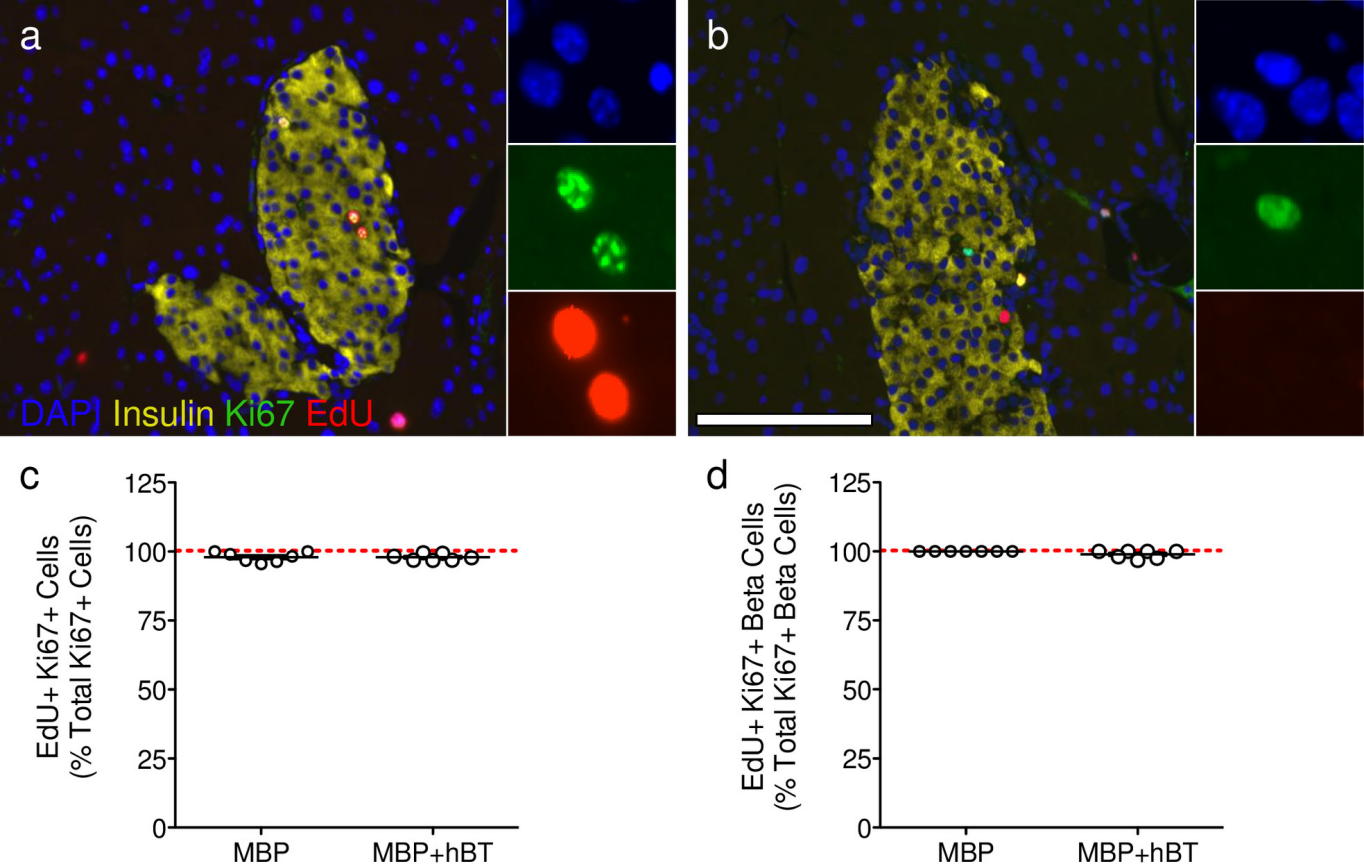
EdU captures all proliferative events detected by Ki67. **(a-b)** Immunostaining by Lab #2 for insulin (yellow), Ki67 (green), EdU (red), and DAPI (blue). Scale bar: 100 μm. Insets demonstrate **(a)** Ki67^+^ EdU^+^ co-positive cells and **(b)** a rare Ki67^+^ EdU- cell. **(c-d)** Quantification of Ki67^+^ cells co-expressing EdU in **(c)** all pancreatic cells and **(d)** β-cells. Data are reported as the mean ± SEM. MBP and MBP+hBT = 7 animals per group.

**Fig 6:**
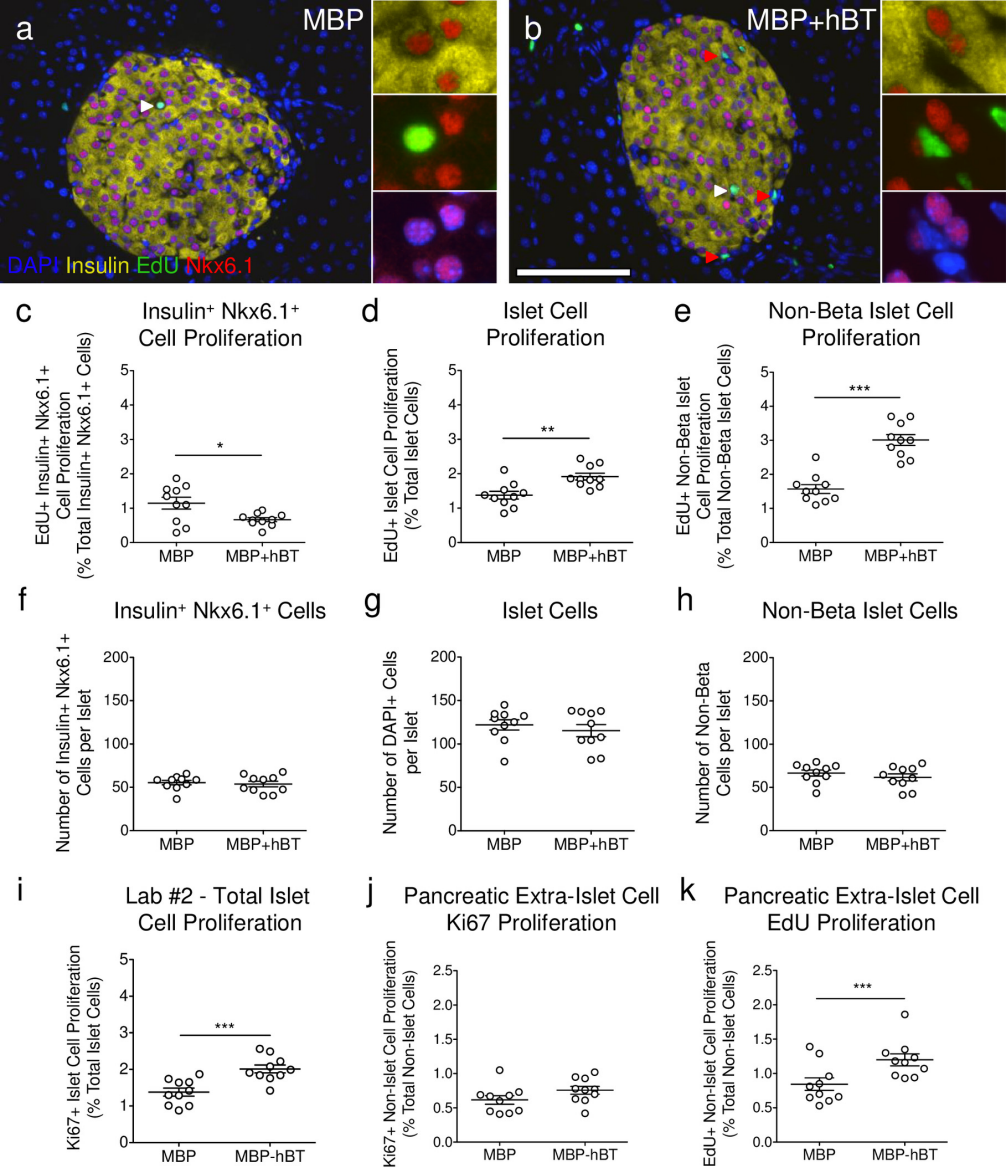
ANGPTL8 treatment in mice increases non-β-cell proliferation. **(a-b)** Staining performed by Lab #2 for insulin (yellow), EdU (green), Nkx6.1 (red), and DAPI (blue) for **(a)** MBP and **(b)** MBP+hBT islets. White arrowheads indicate a proliferating insulin^+^ Nkx6.1^+^ DAPI^+^ cell [inset **(a)**] and red arrowheads indicate a proliferating insulin^-^ Nkx6.1^-^ DAPI^+^ cell [inset **(b)**]. Scale bar: 100 μm. **(c-e)** Quantification of EdU^+^ proliferation for **(c)** β-cells, **(d)** total islet cells, and **(e)** non-β islet cells. **(f-h)** Quantification of **(f)** β-cell number, **(g)** total islet cell number, and **(h)** non-β islet cell number per islet. Islet cells were identified by dilating insulin area by one cell’s diameter and filling all holes within the object. β-cells were identified by Nkx6.1^+^ cells co-localized with DAPI surrounded by insulin. Non-β islet cells were calculated by subtracting the β-cell counts from the total islet cell counts. **(i)** Total islet cell proliferation by Ki67 from original stained slides by Lab #2 examined in Fig 3. **(j-k)** Quantification of pancreatic proliferation by **(j)** Ki67^+^ or **(k)** EdU^+^ (% of total non-islet cells) from original stained slides by Lab #2 examined in Fig 3. Data are mean ± SEM. MBP and MBP+hBT = 10 animals per group. Student’s *t* test was performed. * *p* < 0.05, ** *p* < 0.01, *** *p* < 0.001.

We carried out a collaborative blinded study to test the impact of ANGPTL8 upon β-cell proliferation (Fig 1). Three laboratories (Kushner, Melton, and Yi) participated in the project. The experiment was validated (Fig 2) and performed as described in Fig 3A, followed by blood and pancreatic tissue collection for subsequent analysis. Pancreatic tissue sections were distributed to each laboratory for independent immunohistochemical staining and quantification of β-cell proliferation. After β-cell proliferation analyses were independently completed, the data (Figs 3 and 4) was exchanged between all three groups followed by unblinding [revealing the randomization code (S1 Table)]. Regular conference calls allowed for discussion of experimental results and plans, and additional, unblinded experiments were then performed (Figs 5 and 6) to extend the initial analysis.

### ANGPTL8 treatment in mice potently stimulates dyslipidemia

Previous experiments examined the β-cell response to overexpression of ANGPTL8 DNA in the liver of mice. In these experiments the resulting liver transfection was extremely variable [7], presenting a potential limitation of this method. We therefore used intravenous injection of soluble recombinant ANGPTL8 protein. Producing soluble recombinant ANGPTL8 protein is technically challenging and several expression systems we tried yielded insoluble aggregates (Yi unpublished observation). To circumvent this limitation human ANGPTL8 (hBT) was fused to maltose binding protein (MBP), which can increase soluble expression of heterologous proteins [11, 12]. The resulting MBP+hBT fusion protein was expressed in *E*.*coli* and soluble (Fig 2A and 2B). To test the impact of ANGPTL8 protein, we injected 11ug of MBP alone or MBP+hBT intravenously on day 1 and 2 with concurrent administration of EdU to capture β-cell proliferation (Fig 3A). The mice tolerated ANGPTL8 injection without any obvious distress; body weight, blood glucose, and serum insulin were unaffected by MBP+hBT treatment (Fig 3B–3D, S2 Table). In contrast with prior reports involving long-term sustained ANGPTL8 expression [1, 6, 7], 2-day administration of ANGPTL8 had no effect on triglyceride levels triglyceride levels when measured 24 hours after the second dose (Fig 3E, S2 Table). However, in a separate experiment MBP+hBT injection showed higher serum triglycerides than MBP alone injection after 6 hours (Fig 2C and 2D, S3 Table), demonstrating the bioactivity of MBP+hBT. Thus, fusion of hBT to MBP phenocopies previous studies using various ANGPTL8 expression methods and indicates that treatment with recombinant protein allows for testing of ANGPTL8 effects.

### ANGPTL8 treatment in mice has no significant effect on β-cell proliferation

To assess the effect of ANGPTL8 treatment on β-cell proliferation, each lab quantified β-cell replication in blinded pancreatic samples (Fig 4, S4 Table). We employed multiple markers (Ki67 and EdU) to detect and confirm β-cell replication, and to allow continuous labeling of dividing cells over the period of study. The Kushner group has previously observed that labeling with BrdU does not alter β-cell proliferation [9]. In contrast, Heimberg and colleagues recently reported that thymidine labeling with IdU might decrease β-cell proliferation in 8-week-old mice [13]. Consequently, we first tested if EdU labeling might alter β-cell proliferation in 10-week-old CD1 mice. Buffer injected mice were labeled with or without EdU for 2 days followed by sacrifice. Reassuringly, β-cell proliferation, as measured by the proportion of β-cells that were Ki67^+^ insulin^+^, was unaltered by exposure to EdU for 2 days (Fig 4E–4G, S4 Table). To further control for the use of EdU and maltose binding protein (MBP), we also compared mice injected with buffer or MBP and labeled with EdU for 2 days. No difference in β-cell proliferation (Ki67 or EdU) was observed in buffer versus MBP-treated mice (Fig 4E–4J, S4 Table). Taken together, these studies indicate that neither EdU nor MBP influence β-cell proliferation.

In parallel we carried out blinded studies of β-cell proliferation in ANGPTL8 treated mice. Lab #1 found no difference in β-cell replication between MBP and MBP+hBT treated mice, as quantified by Ki67 (Fig 4E, S4 Table). Similarly, Lab #2 found that ANGPTL8 had no impact on β-cell proliferation as determined by Ki67 expression (Fig 4F, S4 Table). Lab #3 observed a 2-fold increase in β-cell proliferation as determined by Ki67 expression in adjacent slides from the same MBP+hBT treated mice (p<0.001; Fig 4G, S4 Table). Importantly, all three groups were blinded to the identity of the slides. Thus differences in methods used to quantify β-cell proliferation were responsible for the disparate results between research labs.

To further assess the impact of ANGPTL8 on β-cells, we quantified β-cell proliferation using EdU. Quantification of EdU incorporation revealed no effect of ANGPTL8 on β-cell proliferation. Notably, this result was independently obtained by all three labs (Fig 4H–4J, S4 Table), which raises the possibility that results obtained by quantifying β-cell replication using Ki67 could have been influenced by some other contributing factor unique to Lab #3. We then considered the possibility that continuous labeling with EdU might not capture all β-cell proliferative events. If so, EdU incorporation could fail to detect β-cell proliferation that was observed by Lab #3 with Ki67. We carried out extensive quantitative analysis of Ki67 EdU co-positive cells. However, detection of EdU incorporation captured 99.4% of β-cell proliferative events detected by staining for Ki67 (Fig 5, S5 Table). Thus, EdU and Ki67 are equally efficient at detecting β-cell proliferation; very few β-cell proliferative events detected by Ki67 expression failed to be detected by EdU incorporation. These results indicate that the differential β-cell proliferation results obtained with Ki67 and EdU by Lab #3 cannot be explained by inadequate EdU labeling of proliferating β-cells. This raised the possibility that results obtained by quantification of β-cell replication using Ki67 by Lab #3 is perhaps due to the misidentification of cells labeled with insulin and replication markers.

### ANGPTL8 treatment in mice increases non-β-cell proliferation

To further understand the variation in β-cell proliferation between labs, we performed linear regression analysis of the Ki67 and EdU β-cell proliferation data. If EdU and Ki67 both accurately detect β-cell proliferation, as demonstrated by Fig 5 and described by the controls in Fig 3, we would expect a positive correlation between EdU and Ki67 β-cell proliferation. Both Lab #1 and #2 confirmed a positive correlation between EdU and Ki67 β-cell proliferation, while data from Lab #3 showed no correlation (Fig 4K–4M, S4 Table). These results suggest a disconnect between Ki67 and EdU detection by Lab #3 which may result from confounding factors in the quantification of β-cell proliferation. To interrogate potential confounders, Lab #2 performed an analysis with a novel methodology to precisely identify proliferating β-cells and total β-cell number. MBP and MBP+hBT pancreatic sections were stained with EdU and insulin as previously, along with Nkx6.1, a well-defined transcription factor expressed in mature insulin producing β-cells [14]. Immunostaining demonstrated that the vast majority of DAPI^+^ insulin^+^ cells also expressed Nkx6.1, thus confirming the specificity of Nkx6.1 as a marker for β-cells (Fig 6A and 6B). Notably, a sub-population of DAPI^+^ insulin^+^ cells was Nkx6.1^-^, but these cells represent a small minority of the total β-cells and did not contain EdU. Importantly, the nuclear Nkx6.1 staining allowed for precise identification of β-cells and computer-aided β-cell detection using image thresholding and quantitative software without manual counting. These studies confirmed that β-cell proliferation was not increased by ANGPTL8 (Fig 6C, S6 Table). Indeed β-cell proliferation, as quantified by EdU^+^ insulin^+^ Nkx6.1^+^ DAPI^+^ cells, was slightly decreased in ANGPTL8 treated mice.

Next we measured total islet cell proliferation to further examine the impact of ANGPTL8 upon the islet. Total islet cell proliferation in MBP+hBT mice was increased 1.4-fold compared to MBP (Fig 6D, S6 Table). We then quantified proliferation amongst non-β-cell components of the islet by subtracting β-cells from total islet counts. Non-β islet cell proliferation in MBP+hBT mice was increased by ~2-fold compared to MBP alone (*p*<0.001; Fig 6E, S6 Table). This observation indicates that ANGPTL8 stimulated proliferation of non-β-cells within the islet. However, the total number of proliferating cells was still modest. As expected, total islet cells were not increased in our short-term study (Fig 6F–6H, S6 Table). β-cell number per islet was unaltered between MBP and MBP+hBT groups (Fig 6F).

The possibility that intra-islet proliferating cells could contaminate islet counts and prevent precise quantification of replicating β-cells versus intra-islet cells was examined. We measured total islet cell proliferation (Fig 6I) using Ki67 in our original samples described in Fig 4 and compared the result with total β-cell proliferation determined using Ki67 from each lab (Fig 4E–4G, S4 and S7 Tables). Interestingly, total islet cell proliferation in MBP+hBT treated mice was very similar to the β-cell proliferation counts of Lab #3 (2.01% islet cell vs 1.65% β-cell) but not that of the Labs #1 and 2 (1.35% Lab #1 β-cell, 0.36% Lab #2 β-cell) (Figs 4E–4G and 6I, S4 and S7 Tables). This similarity indicates that variable β-cell proliferation results amongst research groups could result from differences in identifying and counting β-cells within the larger population of proliferating islet cells. We extended our proliferation analysis to include total pancreatic proliferation and found that ANGPTL8 significantly increased extra-islet cell proliferation, compared to MBP alone as measured by EdU, with a weak trend (p = 0.11) towards increased proliferation by Ki67 (Fig 6J and 6K, S8 Table). Taken together, these findings indicate that ANGPTL8 signals may stimulate non-β-cell proliferation within the islet and surrounding exocrine tissue and this may account for some of the reported discrepancies. Even so, there is no evidence in these experiments to support the claim of a 17-fold increase in β-cell replication by ANGPTL8 [4]. Instead, the present studies further support previous observations that ANGPTL8 does not stimulate robust β-cell proliferation [7].

### ANGPTL8 treatment in mice increases hematopoietic-derived cell proliferation

**Fig 7:**
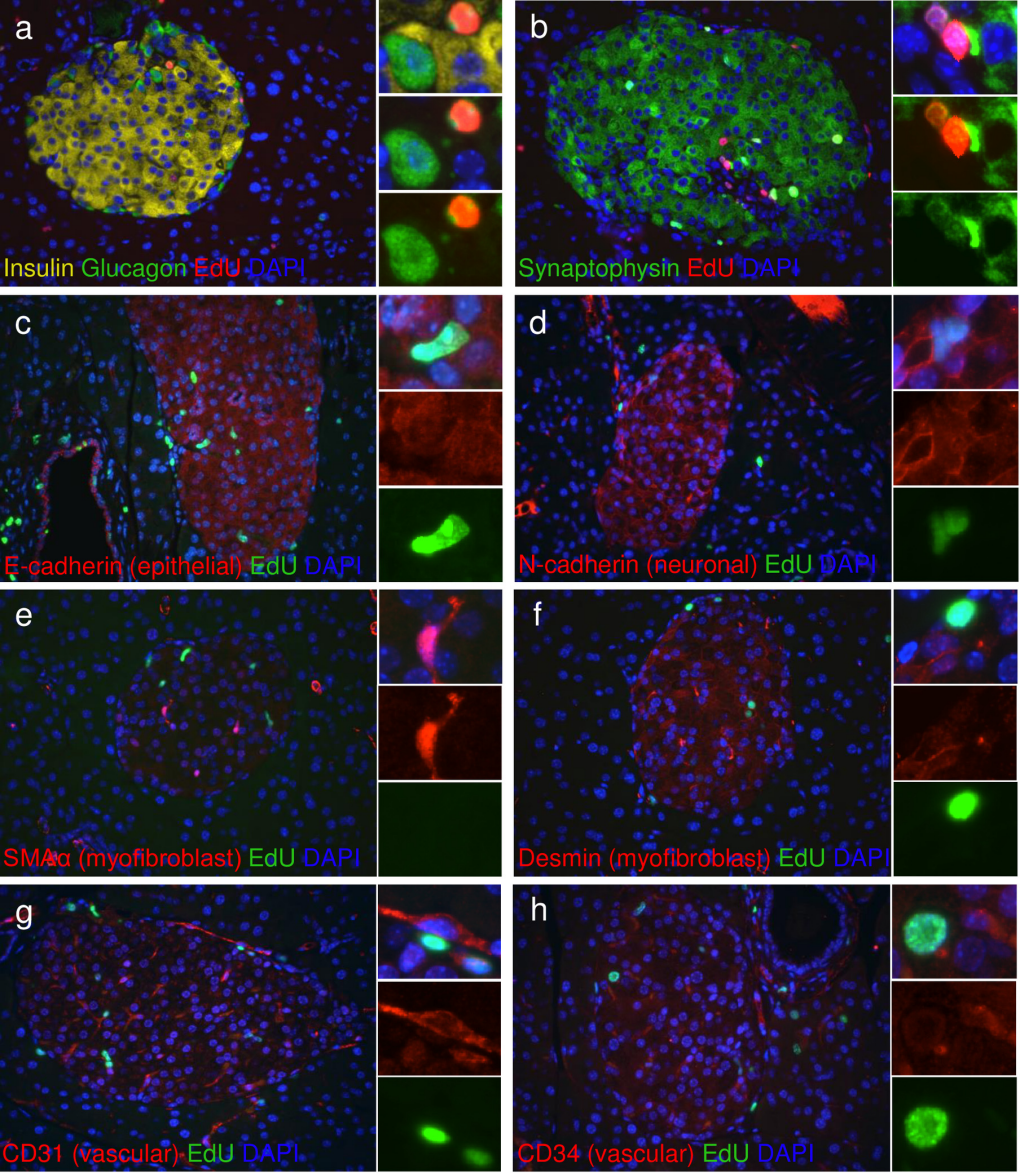
Highly proliferative cells in islets of ANGPTL8 treated mice are not glucagon, endocrine, epithelial, neuronal, myofibroblast, or vascular cells. **(a)** Immunostaining for insulin (yellow), glucagon (green), EdU (red) and DAPI (blue). **(b)** Immunostaining for synaptophysin (green), EdU (red), and DAPI (blue). **(c-h)** Immunostaining for EdU (green) and DAPI (blue) with various makers (red); **(c)** E-cadherin, **(d)** N-cadherin, **(e)** smooth muscle actin α (SMAα), **(f)** desmin, **(g)** CD31, **(h)** CD34. Insets indicate EdU positive cells that do not express glucagon, synaptophysin, E-cadherin, N-cadherin, SMAα, desmin, CD31, or CD34. Scale bar = 100 μm.

**Fig 8:**
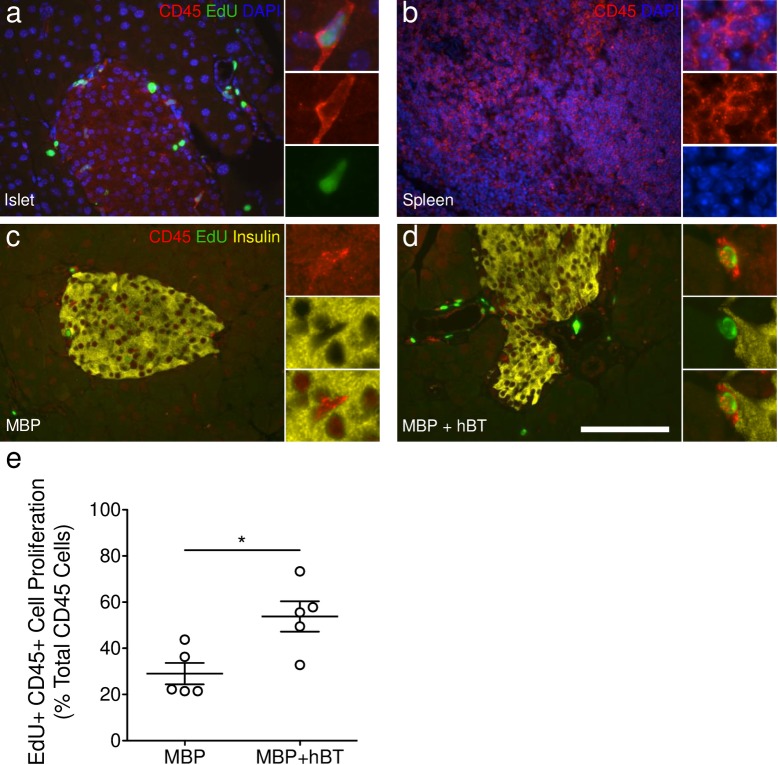
ANGPTL8 treatment in mice increases hematopoietic-derived cell proliferation. **(a-d)** Staining for CD45 (red), EdU (green), insulin (yellow), and DAPI (blue) in **(a)** pancreatic islets, in **(b)** spleen used as a positive control, and in islets of **(c)** MBP or **(d)** MBP+hBT treated mice. **(e)** Quantification of CD45^+^ intra- and peri-islet cell proliferation. Intra- and peri-islet cells were identified by dilating insulin area by one cell’s diameter and filling all holes within the object. Scale bar = 100 μm. Student’s *t* test was performed. * *p* < 0.05.

**Fig 9:**
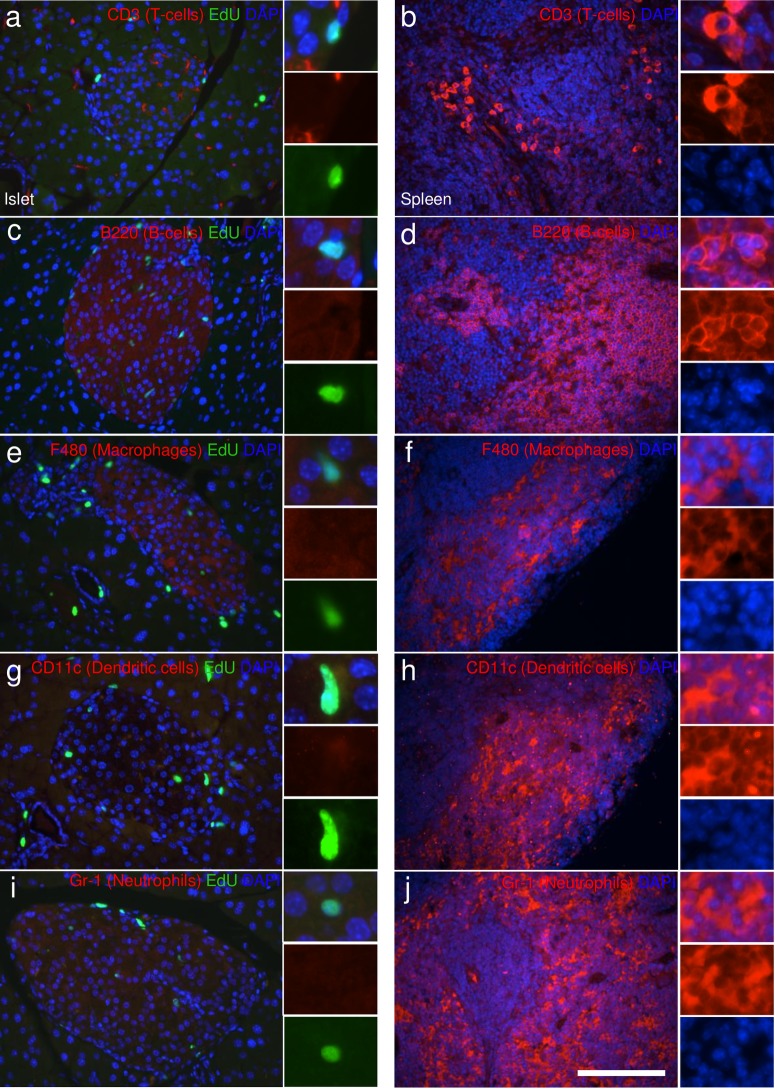
Highly proliferative cells in islets of ANGPTL8 treated mice are not immune related cells. Immunostaining for EdU (green) and DAPI (blue), with various markers for immune-related cells (red) in **(a,c,e,g,i)** pancreatic islets or in **(b,d,f,h,j)** the spleen used as a positive control. **(a-b)** CD3 (T-cells), **(c-d)** B220 (B-Cells), **(e-f)** F480 (macrophages), **(g-h)** CD11c (dendritic cells), and **(i- j)** Gr-1 (Neutrophils). Insets indicate an EdU^+^ replicating cell that does not express CD3, B220, F480, CD11c, or Gr-1. Scale bar = 100 μm.

Our studies show that ANGPTL8 stimulates proliferation of a population of non-insulin containing cells within the islet. We performed immunohistochemical stains to identify the replicating islet cell population and that very few glucagon cells had undergone cell division (Fig 7A). Nor did the EdU positive cells within and around islets express synaptophysin, a pan-endocrine marker, suggesting that the replicating cell population in ANGPTL8 treated mice is not hormone containing (Fig 7B). Furthermore, the EdU^+^ cells within the islet did not express epithelial or neuronal cadherins, nor the myofibroblast markers smooth muscle actin alpha and desmin (Fig 7C–7F). To test for potential infiltrating bone marrow derived cells, we stained for the hematopoietic cell marker CD45 and observed a high frequency of EdU^+^ cells expressing CD45 within the islet and the surrounding acinar tissue (Fig 8A and 8B). Quantification demonstrated that CD45^+^ cell proliferation within islets was significantly increased in MBP+hBT treated mice (Fig 8C–8E). CD45^+^ EdU^+^ cells accounted for some but not all of the intra- and peri-islet cell proliferation. Vascular cells identified by CD31 or CD34 were rarely found to contain EdU (Fig 7G and 7H). Examining lymphocyte markers within the islets of ANGPLT8 treated mice, we found no expression of CD3 (T-cell) or B220 (B-cell) (Fig 9A–9D), although T-cells were commonly observed in large blood vessels (data not shown). Furthermore EdU^+^ cells within the islet did not express markers of antigen presenting cells (macrophages, dendritic cells) nor neutrophils (Fig 9E–9J). These results suggest highly proliferative islet cells are non-endocrine, bone marrow-derived cells that do not express markers of mature vascular or immune cells. Thus, ANGPTL8 treatment stimulates proliferation and possibly migration of CD45^+^ hematopoietic-derived cells.

## Discussion

Historically, discrepant observations between labs have been difficult to resolve. Differing reagents and methodologies may produce different biases. Sometimes these differences fade with the passing of time, and researchers, especially those new to the field, find the literature confusing and insecure. We aimed instead to work together to settle the record surrounding the betatrophin hypothesis by performing a collaborative blinded study. The independent analysis from all three different labs shows that ANGPTL8 does not stimulate robust β-cell proliferation. While we cannot rule out a modest effect of less than 2 fold on replication, there is no evidence to support any dramatic effect on beta cell replication. We did find that ANGPTL8 treatment increased proliferation of CD45^+^ hematopoietic-derived cells within the islet, and also cells in the surrounding exocrine tissue.

The strength of this collaborative approach derives from the independent assessment of β-cell proliferation in an unbiased manner. This strategy provides a level of confidence and rigor typically employed in clinical trials, but rarely so in basic science experiments. Through this process we collectively reached the conclusion that ANGPTL8 does not stimulate robust β-cell proliferation in mice, confirming previous observations [6, 7].

We encountered two limitations during the course our study. First, the half-life of MBP+hBT in serum was lower than expected as we were unable to detect a significant increase in serum ANGPTL8 twenty-four hours after the second dose. Regardless, we detected bioactivity of MBP+hBT in a time course analysis of ANGPTL8 effects on serum triglycerides (Fig 2B). Injection of MBP+hBT protein increased serum triglycerides by 27% at 6 hours, which was equivalent to previous experiments that observed a 28% increase in serum triglycerides (Cox et al. Diabetologia, 2015) with sustained low dose expression of ANGPTL8 via plasmid DNA injection. Further increases in triglycerides may have been limited by the short half-life of MBP+hBT or by the capacity for ANGPTL8-induced triglyceride mobilization. Secondly, we discovered endotoxin in the protein preparations. Although, endotoxin could be responsible for migration and proliferation of CD45^+^ cells, there are currently no studies to indicate whether endotoxin has any influence on β-cell proliferation.

The main challenge in analyzing beta cell replication is the very low number of dividing cells; so many sections and cells have to be counted to approach a solid conclusion. Notably, we observed variability in β-cell proliferation counting between labs. One potential source of variability may be differences in antibodies and reagents used to detect proliferation. For example, Lab #2 used a mouse Ki67 primary antibody that required an anti-mouse secondary antibody, which produced non-specific staining that may be counted as positive staining. This was resolved by manual counting to ensure that only Ki67^+^ insulin^+^ DAPI^+^ cells were counted. Another possibility is that EdU may decrease or fail to detect Ki67 β-cell proliferative events. However, we show here and elsewhere, that EdU captures 99.4% of β-cell proliferative events detected by Ki67 and thymidine analogs do not alter Ki67^+^ β-cell proliferation (Fig 3, S4 Table; [9]), in contrast to previous reports [13]. Comparing methods between labs, we speculate that two key differences may have influenced β-cell quantification. First, image acquisition was performed with objectives of different magnification [10x (NA = 0.3), 20x (NA = 0.8) versus 40x (NA = 0.75)]. Lower power objectives provide less resolution, limiting the ability to differentiate β-cells from non-β-cells. Secondly, manual counting of DAPI nuclei surrounded by insulin may be subjected to user bias while computer-aided counting is objective. Computer-aided identification of DAPI nuclei in contact with insulin resulted in a slight over count of the total number of β-cells compared to manual hand counting of β-cells. A second approach used Nkx6.1, a marker of mature insulin-expressing β-cells [14], along with DAPI and insulin. Computer-aided β-cell counts using Nkx6.1 were comparable to manual counting, noting that only a small percentage of cells that were insulin^+^ DAPI^+^ Nkx6.1^-^ might be considered β-cells based on manual counting of insulin and DAPI alone, although these insulin^+^ DAPI^+^ Nkx6.1^-^ were very rarely EdU^+^. In all, the variability observed between groups may result from the detection and counting of cells, which was further obscured by an increased presence of proliferating CD45^+^ cells that might have been quantified as proliferating β-cells. Regardless of the method used, the same conclusion was obtained: ANGPTL8 does not stimulate robust β-cell proliferation.

The field of β-cell biology is dependent on precise quantification of β-cells to accurately assess the impact of various interventions on β-cell expansion. Because β-cells divide so rarely, the quantification is challenging, yet merely a 2x increase in proliferation rates could be clinically relevant. The lack of standardization for β-cell quantification in the field is demonstrated by the current study wherein three labs used three different approaches to quantify β-cells. This indicates that similar variation likely exists within the β-cell biology literature. Despite the variation observed in the current report, analysis of three independent data sets point to the strength of a blinded collaborative approach to resolve a controversial finding.

It was reported that overexpression of ANGPTL8 DNA in the liver of mice induced a dramatic increase in β-cell proliferation [4]. It is possible that the 17-fold increase in β-cell proliferation may have been observed due to varied methodology for the quantification of β-cells. Here we observed a significant increase in CD45^+^ hematopoietic-derived cell proliferation throughout the islet, which might have been confused with β-cells, leading to a perceived increase in β-cell proliferation. Grayburn and colleagues recently reported that ANGPTL8 increased β-cell proliferation dramatically by ~7-fold in 3-month-old rats (~0% in control versus 7.24% in *ANGPTL8* treated) using ultrasound-targeted microbubble destruction to deliver human *ANGPTL8* plasmids to the rat pancreas [8]. It is possible these findings result from species-specific differences or the method of ANGPTL8 administration.

One of the two main conclusions of the original paper describing the betatrophin hypothesis [4] needs to be withdrawn. ANGPTL8 was proposed to underlie the β-cell mitogenic signals that resulted in β-cell expansion in response to insulin resistance. While the current experiment was performed with a different reagent (protein versus plasmid DNA) and we have uncovered difficulties quantifying β-cells, we can no longer support the claim that ANGPTL8 has a dramatic effect on β-cell proliferation. The other principal finding, namely the effect of the insulin antagonist S961 in stimulating β-cell proliferation (4), has been repeated by several groups (6,14). Thus, the outstanding biological and clinically relevant question now is: what are the signals that stimulate β-cell proliferation in S961-treated insulin resistant mice?

In summary, a collaborative group effort is a very effective method to settle a controversy. Experimental conclusions can vary through intra-observer variability (for example, an inconsistent counting methodology) or inter-observer variability (differing methodologies or true biological variability due to the presence of some unrecognized and therefore uncontrolled biological variable). Our study highlights the challenges for identifying β-cells (staining methods; low-power versus high-power image acquisition; manual versus computer-aided quantification) and we propose that standard practices for β-cell quantification be developed. Indeed, this may include the standardized use of 2 differing protocols to ensure further accuracy since, for such important results, it should be required to have multiple levels of evidence for any conclusion. Through strong collaboration, we have resolved the betatrophin controversy and concluded that ANGPTL8 does not induce robust β-cell proliferation. Insulin resistance remains a powerful model for β-cell expansion and identification of the underlying signals, while challenging, will be important to uncover targets for β-cell regenerative therapies in diabetes.

## Supporting Information

S1 Table Code for MBP and MBP+hBT injections. (XLSX)

S2 Table Individual physiologic data. Measurements were recorded from 10-week-old CD1 male mice injected with buffer (with or without EdU), maltose binding protein (MBP; control) or MBP-human betatrophin (hBT) fusion protein for two days and sacrificed on day three. Initial and final body weight (g), initial and final random fed blood glucose (mg/dl), random fed serum insulin (ng/ml) and human or mouse ANGPTL8 (ng/ml; pg/ml), and random fed serum triglyceride (TG; mg/dl).
(XLSX)

S3 Table In a separate eperiment, acute administration of ANGPTL8 transiently increased serum triglycerides. Measurements were recorded from mice injected with maltose binding protein (MBP; control), MBP-human betatrophin (hBT) fusion protein, or various concentrations of lipase inhibitor (increases serum triglyceride) and followed for two days. Final body weight (g) and random fed blood glucose (mg/dl). Serum triglycerides (mg/dl) were measured at 0, 1, 3, 6, and 24 hours after treatment.
(XLSX)

S4 Table Individual data for β-cell proliferation analysis from all three labs for ANGPTL8 treatment studies. (top panel) Total β-cells counted for Ki67, total Ki67^+^ insulin^+^ DAPI^+^ cells, and Ki67^+^ β-cells (% of total insulin+ cells) for the tail pancreas. (bottom panel) Total β-cells counted, total EdU^+^ insulin^+^ DAPI^+^ cells, and EdU+ β-cells (% of total insulin^+^ cells). 10-week-old CD1 male mice injected with buffer with or without EdU, or injected with MBP or MBP+hBT receiving EdU.
(XLSX)

S5 Table Individual data for Ki67, EdU co-expressing total cell and β-cell analysis. Measurements were recorded from 10-week-old CD1 male mice injected with maltose binding protein (MBP; control) or MBP-human betatrophin (hBT) fusion protein for two days and sacrificed on day three. Quantification of Ki67^+^ EdU^+^ cells as a percenatge of total cells or β-cells.
(XLSX)

S6 Table Individual data for islet cell, β-cell, and non-β islet cell counts & proliferation using Nkx6.1 as a marker for β-cells. Measurements were recorded from 10-week-old CD1 male mice injected with maltose binding protein (MBP; control) or MBP-human betatrophin (hBT) fusion protein for two days and sacrificed on day three. Islet cells were identified by dilating the insulin area by one cell’s diameter and then filling all holes within the region of the object. β-cells were identified by Nkx6.1^+^ cells co-localized with DAPI surrounded by insulin. Non-β islet cells were calculated by subtracting the β-cell counts from the total islet cell counts.
(XLSX)

S7 Table Individual data for islet cell Ki67 proliferation based on stained slides described in Fig 3. Measurements were recorded from 10-week-old CD1 male mice injected with maltose binding protein (MBP; control) or MBP-human betatrophin (hBT) fusion protein for two days and sacrificed on day three. Islet cells were identified by dilating the insulin area by one cell’s diameter and then filling all holes within the region of the object. Quantification of total Ki67 proliferation (% of total DAPI^+^ islet cells).
(XLSX)

S8 Table Individual data for exocrine cell proliferation for ANGPTL8 treatment studies. Measurements were recorded from 10-week-old CD1 male mice injected with maltose binding protein (MBP; control) or MBP-human betatrophin (hBT) fusion protein for two days and sacrificed on day three. Quantification of pancreatic proliferation by Ki67 or EdU (% of total exocrine cells). Exocrine cells were calculated by subtracting total DAPI^+^ islet cells from all DAPI cells.
(XLSX)
